# Bacterial vaginosis in pregnant adolescents: proinflammatory cytokine and bacterial sialidase profile. Cross-sectional study: Vaginose bacteriana em gestantes adolescentes: perfil de citocinas proinflamatórias e sialidases bacterianas. Estudo transversal

**DOI:** 10.1590/1516-3180.2014.9182710

**Published:** 2015-10-09

**Authors:** Carolina Sanitá Tafner Ferreira, Camila Marconi, Cristina Maria de Lima Garcia Parada, Marli Teresinha Cassamassimo Duarte, Ana Paula Oliveira Gonçalves, Marilza Vieira Cunha Rudge, Márcia Guimarães da Silva

**Affiliations:** I BSc, Master's Student, Department of Pathology, Faculdade de Medicina de Botucatu (FMB), Universidade Estadual Paulista (Unesp), Botucatu, São Paulo, Brazil.; II BSc, MSc, PhD. Postdoctoral Fellow. Department of Pathology, Faculdade de Medicina de Botucatu (FMB), Universidade Estadual Paulista (Unesp), Botucatu, São Paulo, Brazil.; III BSN, MSc, PhD. Adjunct Professor, Department of Nursing, Faculdade de Medicina de Botucatu (FMB), Universidade Estadual Paulista (Unesp), Botucatu, São Paulo, Brazil.; IV BSN, MSc, PhD. Assistant Professor, Department of Nursing, Faculdade de Medicina de Botucatu (FMB), Universidade Estadual Paulista (Unesp), Botucatu, São Paulo, Brazil; V BSN, MSc. Assistant Professor, Department of Nursing, Universidade Federal do Pará (UFPA), Belém, Pará, Brazil.; VI MD, MSc, PhD. Titular Professor, Department of Gynecology and Obstetrics, Faculdade de Medicina de Botucatu (FMB), Universidade Estadual Paulista (Unesp), Botucatu, São Paulo, Brazil.; VII BSc, MSc, PhD. Assistant Professor, Department of Pathology, Faculdade de Medicina de Botucatu (FMB), Universidade Estadual Paulista (Unesp), Botucatu, São Paulo, Brazil.

**Keywords:** Pregnancy, Adolescent, Vaginosis, bacterial, Cytokines, Neuraminidase.

## Abstract

**CONTEXT AND OBJECTIVE:**

Bacterial vaginosis occurs frequently in pregnancy and increases susceptibility to sexually transmitted infections (STI). Considering that adolescents are disproportionally affected by STI, the aim of this study was to evaluate the cervicovaginal levels of interleukin (IL)-1 beta, IL-6, IL-8 and bacterial sialidase in pregnant adolescents with bacterial vaginosis.

**DESIGN AND SETTING:**

Cross-sectional study at mother and child referral units in Belém, Pará, Brazil.

**METHODS:**

Vaginal samples from 168 pregnant adolescents enrolled were tested for trichomoniasis and candidiasis. Their vaginal microbiota was classified according to the Nugent criteria (1991) as normal, intermediate or bacterial vaginosis. Cervical infection due to *Chlamydia trachomatis *and *Neisseria gonorrhoeae* was also assessed. Cytokine and sialidase levels were measured, respectively, using enzyme-linked immunosorbent assays and MUAN conversion in cervicovaginal lavages. Forty-eight adolescents (28.6%) were excluded because they tested positive for some of the infections investigated. The remaining 120 adolescents were grouped according to vaginal flora type: normal (n = 68) or bacterial vaginosis (n = 52). Their cytokine and sialidase levels were compared between the groups using the Mann-Whitney test (P < 0.05).

**RESULTS:**

The pregnant adolescents with bacterial vaginosis had higher levels of IL-1 beta, IL-6 and IL-8 (P < 0.05). Sialidase was solely detected in 35 adolescents (67.2%) with bacterial vaginosis.

**CONCLUSIONS:**

Not only IL-1 beta and sialidase levels, but also IL-6 and IL-8 levels are higher in pregnant adolescents with bacterial vaginosis, thus indicating that this condition elicits a more pronounced inflammatory response in this population, which potentially increases vulnerability to STI acquisition.

## RESUMO

**CONTEXTO E OBJETIVO:**

A vaginose bacteriana é uma condição, comum em gestantes, que aumenta a susceptibilidade a infecções sexualmente transmissíveis (IST). Considerando que adolescentes são desproporcionalmente afetadas por IST, o objetivo deste estudo foi avaliar os níveis cervicovaginais de interleucina (IL)-1 beta, IL-6, IL-8 e sialidases bacterianas em gestantes adolescentes com vaginose bacteriana. DESENHO DO ESTUDO E LOCAL: Estudo transversal em Unidade de Referência Materno Infantil

(UREMIA), Belém, Pará, Brasil.

**MéTODOS:**

Amostras vaginais das 168 gestantes adolescentes incluídas foram testadas para tricomoníase e candidíase e a microbiota vaginal foi classificada em normal, intermediária e vaginose bacteriana, segundo os critérios de Nugent (1991). infecções cervicais por *Chlamydia trachomatis *e *Neisseria gonorrhoeae* também foram avaliadas. Os níveis de citocinas e sialidades foram quantificados, respectivamente, por método imunoenzimático e pela conversão do MUAN nos lavados cervicovaginais. Foram excluídas 48 (28,6%) adolescentes positivas para alguma das infecções investigadas. As 120 gestantes remanescentes foram agrupadas de acordo com o padrão de flora vaginal em: normal (n = 68) e vaginose bacteriana (n = 52). Níveis de citocinas e sialidases foram comparados pelo teste de Mann-Whitney, P < 0,05.

**RESULTADOS:**

As gestantes adolescentes com vaginose bacteriana entre os grupos apresentaram níveis aumentados de IL-1 beta, IL-6 and IL-8 (P < 0,05). Sialidases foram exclusivamente detectadas em 35 (67,2%) adolescentes com vaginose bacteriana.

**CONCLUSÕES:**

Não apenas a IL-1 beta e as sialidases estão aumentadas em gestantes adolescentes com vaginose bacteriana, mas também IL-6 e IL-8, indicando resposta inflamatória mais pronunciada dessa alteração de microbiota nesta população, potencializando a vulnerabilidade à aquisição de IST.

## INTRODUCTION

Bacterial vaginosis is the most common type of abnormal vaginal flora and it is frequent in both pregnancy and adolescence.^1^ ^3^ In bacterial vaginosis, vaginal lactobacilli are replaced by other bacterial species, mostly anaerobes.^4^ It has been consistently shown in the literature that bacterial vaginosis is strongly associated with increased risk of preterm delivery and acquisition of sexually transmitted infections (STIs).^1^ ^5^ ^7 ^The mechanisms underlying the associations between bacterial vaginosis and poor pregnancy outcomes and higher vulnerability to STIs remain to be addressed, but it is well established that alterations to vaginal immunity play a crucial role in them.^8^ ^9^

In fact, pregnancy and adolescence are both conditions associated with changes to the levels of immune mediators in the lower genital tract.^10^ ^12 ^Typically, bacterial vaginosis is associated with increased cervicovaginal levels of interleukin (IL)-1 beta, but in pregnant women the levels of IL-6 and IL-8 are also elevated in the presence of bacterial vaginosis.^13^ ^16 ^There is still a lack of information in the literature regarding the immune response to bacterial vaginosis in adolescents. However, it has already been demonstrated that the vaginal fluids of adolescents present altered levels of several immune mediators and show lower antimicrobial activity *in vitro*, in comparison with adults.^12^ These findings are particularly important given that this population is disproportionally more affected by STIs.^17^

Higher vaginal sialidase levels are another factor associated with increased vulnerability to STIs during bacterial vaginosis. Bacterial sialidase is capable of degrading local immunoglobulins and consequently compromising the local immune defense.^18^ Additionally, elevated vaginal sialidase levels in early pregnancy has already been correlated with elevated risk of occurrence of spontaneous preterm labor.^19^

Considering the importance of balanced vaginal immunity for protecting the lower genital tract against STIs, better understanding of the local response to bacterial vaginosis in pregnant adolescents may contribute towards new strategies for preventing infection in this highly vulnerable population. 

## OBJECTIVE

The objective of the present study was to evaluate the cervicovaginal levels of the proinflammatory cytokines IL-1 beta, IL-6 and IL-8 and bacterial sialidase in pregnant adolescents with bacterial vaginosis.

## METHODS

Between 2009 and 2011, a total of 168 pregnant adolescents were recruited for this cross-sectional study in outpatient clinics for obstetric care for adolescents in the metropolitan area of Belém, Pará, northern region of Brazil. The ethics committee of Universidade Federal do Pará (#002/2009) approved the study. All the participants were accompanied by a family member or partner who was older than 18 years and signed a written consent statement. 

The inclusion criteria were that the patient needed to be pregnant, younger than 19 years and HIV-negative; and to have not used antibiotics recently (last 30 days), to have not had sexual intercourse over the last three days and to have undergone vaginal ultrasound examination within the last three days. All the adolescents answered a standardized questionnaire in order to obtain sociodemographic and behavioral data and underwent pelvic examination for cervicovaginal sampling. 

After inserting a non-moisturized speculum, vaginal pH was ascertained using commercial pH strips (Merck, Darmstadt, Germany) and the whiff test, performed by adding 10% KOH (potassium hydroxide) to the samples. Swabs were taken from the vaginal wall and smeared on microscope slides for Gram staining and classification of the vaginal flora in accordance with the scoring system proposed by Nugent et al.,^20^ as normal (0-3), intermediate (4-6) or bacterial vaginosis (7-10). 

The diagnosis of bacterial vaginosis was based on Nugent scores, regardless of the vaginal pH and whiff test. Gram-stained smears were also used for diagnosing candidiasis when *Candida *sp. hyphae were detected. Additionally, samples taken from the vaginal vault were cultured in Diamond's liquid medium for detection of *Trichomonas vaginalis*. Endocervical samples were also obtained for *Neisseria gonorrhoeae *and *Chlamydia trachomatis *detection by means of, respectively, culturing in Thayer-Martin and the polymerase chain reaction (PCR). 

Cultures for *Neisseria gonorrhoeae* were incubated at 37 °C for up to 48 hours. For *Chlamydia trachomatis* PCR, two sets of primers were used: CTP1 (5'-TAG TAA CTG CCA CTT CAT CA-3') and CTP2 (5'-TTC CCC TTG TAA TTC GTT GC-3'); or PL6.1 (5'-AGA GTA CAT CGG TCA ACG A-3') and PL62 (5'-TCA CAG CGG TTG CTC GAA GCA-3'),^21^ resulting in product sizes of 201 bp and 130 bp, respectively. Reactions were performed using GoTaq Green Master Mix (Promega, Madison, WI, USA) and 4 µl of DNA template with the following cycling: 95 °C for 5 min followed by 40 repetitions of 95 °C for 1 min, 55 °C for 1 min and 72 °C for 1.5 min, and finally, 72 °C for 5 min (Mastercycler, Eppendorf, Germany).

Cervicovaginal lavages were performed using 3 ml of sterile 0.9% saline solution. The total volume was recovered using a plastic pipette, immediately refrigerated and then transported to the laboratory within 4 hours. After centrifugation at 800 x g for 10 minutes, the supernatant was used in the enzyme-linked immunosorbent assay (ELISA) to determine the levels of the proinflammatory cytokines IL-1 beta, IL-6 and IL-8 (DuoSet Kits, R&D Systems, Minneapolis, MN, USA), in accordance with the manufacturer's instructions. Supernatants of the lavages were also acetylneuraminic acid (MUAN) (Sigma-Aldrich, St. Louis, MO, USA), in accordance with methods detailed previously.^13^ ^15^ In both the cytokine and the sialidase assays, samples were tested in duplicates and, if the measured concentration was above the standard curve, the samples were diluted and retested. The intra and interassay variability rates were < 11.0%. The minimum detectable levels for IL-1 beta, IL-6, IL-8 and sialidase assays were, respectively, 0.1 pg/ml, 1.1 pg/ml, 15.9 pg/ml and 0.2 ng/ml. 

Out of the 168 adolescents initially recruited, those who tested positive for *C. trachomatis *(n = 28; 16.7%), trichomoniasis (n = 5; 3.0%) or candidiasis (n = 12; 7.1%) or presented intermediate flora (n = 3; 1.8%) were excluded from the analysis. The remaining 120 adolescents who presented all the criteria for enrollment were divided into two groups according to the classification of their vaginal flora as normal (n = 68; 56.7%) or bacterial vaginosis (n = 52; 43.3%). 

Comparisons of discrete and continuous variables between the groups were made respectively using the chi-square test or Fisher's exact test and the nonparametric Mann-Whitney test. Cytokine and sialidase levels were compared using the MannWhitney test. All analyses were performed using the GraphPad Prism 5.0 software (GraphPad, CA, USA), considering P < 0.05 to be statistically significant. Although no minimum sample size was calculated during the study design phase, a post-hoc analysis showed that the sample had a minimum test power of 85%, considering the IL-1 beta and IL-8 levels found in the cervicovaginal samples from subjects with normal flora and bacterial vaginosis.

## RESULTS

The sociodemographic, behavioral and clinical characteristics of the 120 adolescents enrolled are shown in Table 1. Most of them reported that they were married or cohabiting with a partner (n = 64/102; 62.7%), did not have a paid job (n = 90/100; 90.0%) and had not had any STI diagnosed previously (n = 80/87; 92.0%). The gestational age at enrollment and all the other characteristics did not differ between the groups (P > 0.05) except for vaginal pH (P < 0.0001) and the number of whiff-positive samples (P < 0.0001), which were significantly higher in individuals with bacterial vaginosis than in those with normal flora.

**Figure 1:**
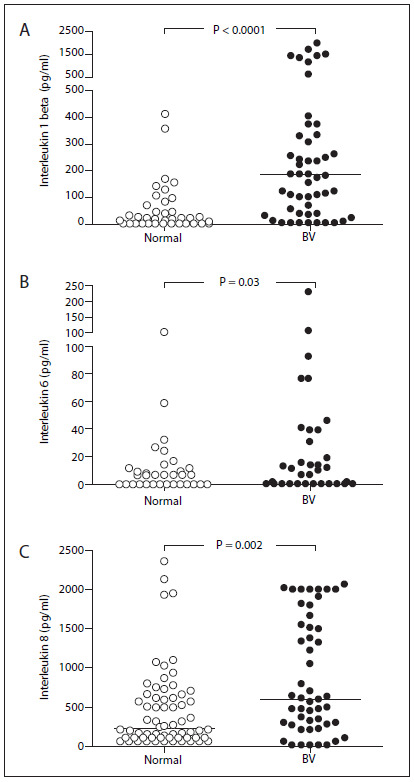
Levels of the proinflammatory cytokines interleukin 1 beta (A), interleukin 6 (B) and interleukin 9 (C) in the cervicovaginal samples from 68 pregnant adolescents with bacterial vaginosis (BV) and 52 with normal vaginal flora. Horizontal bars represent the median (pg/ml).

The results from cytokine assays, with the measured levels of IL-1 beta, IL-6 and IL-8 are shown in Figure 1. The IL-1 beta level was above the detection limit of the assay in 96 cervicovaginal samples (80.0%), while IL-6 and IL-8 levels were determined in respectively 42 (35.0%) and 111 (92.5%) of the 120 samples. The pregnant adolescents with bacterial vaginosis had higher IL-1 beta levels (median 176.7 pg/ml; range 0.0 - 2009.0) in the cervicovaginal samples than did those with normal vaginal flora (median 4.3 pg/ml; range 0.0 - 410.4) (P < 0.0001). IL-6 levels were also significantly higher in individuals with bacterial vaginosis (median 0.0 pg/ml; range 0.0 - 234.6) than in those with normal flora (median 0.0 pg/ml; range 0.0 - 100.1) (P = 0.03). Similarly to IL-1 beta and IL-6, the IL-8 levels were higher in individuals with bacterial vaginosis (median 595.1 pg/ml; range 0.0 - 2070.0) than in those with normal flora (median 215.7 pg/ml; range 0.0 - 2358.0) (P = 0.002). 

Bacterial sialidase was not detected in samples from pregnant adolescents with normal flora. However, a total of 35 (67.2%) of the 52 adolescents with bacterial vaginosis had detectable cervicovaginal levels of this enzyme, with a median concentration of 10.3 ng/ml (range 0.0 - 163.3 ng/ml ).

## DISCUSSION

In this cross-sectional study, we determined the cervicovaginal levels of proinflammatory cytokines and sialidase in pregnant adolescents with bacterial vaginosis living in the northern region of Brazil and compared these levels with those with normal *lactobacilli*-dominated flora. To our knowledge, this is the first report on the inflammatory response to bacterial vaginosis among pregnant adolescents. 

We are aware of the limitation that this study presents through not having an initial estimate for the sample size. However, posthoc analysis produced a satisfactory power test based on the levels of the two cytokines quantified. Therefore, we believe that this limitation does not hamper the findings shown here.

**Table 1:** Sociodemographic, behavioral and clinical characteristics of used to determine the sialidase levels by means of conversion the 120 pregnant adolescents included in the study. Values shown as of the fluorogenic substrate 2-(4-methylumbelliferyl)-a-D-N-median (range) or n/total number (%)

Characteristics^*^	Normal (n = 68)	Bacterial vaginosis (n = 52)	P
Age (years)	16 (13-19)	16 (13-19)	0.73^†^
Gestational age	18 weeks 1 day (8 weeks - 4 weeks 1 day)	16 weeks 1 day (7 weeks - 35 weeks 2 days)	0.10
Marital status			
Single	19/57 (33.3)	19/45 (42.2)	
Married or cohabiting	38/57 (66.7)	26/45 (57.8)	0.36^‡^
Education (years at school)	6.5 (0-12)	7 (1-11)	0.88^†^
Full or part-time paid job	5/56 (8.9)	5/44 (11.4)	0.74^§^
History of previous STI	5/55 (9.1)	2/32 (6.2)	1.00^§^
Regular use of condoms	20/56 (35.7)	16/42 (38.1)	1.00^‡^
Vaginal pH	4.4 (4.0-5.0)	5,0 (4.0-5.3)	< 0.0001^†^
Positive whiff test	8/54 (14.8)	39/43 (90.7)	< 0.0001^§^

*Total number of women may vary among the categories, since some of the data were unavailable or the patient refused to answer; STI = sexually transmitted infection; ^†^Mann-Whitney test; ^‡^chi-square test; ^§^Fisher exact test; P < 0.05.

Recent studies have pointed out that vaginal immunity is compromised by disrupted vaginal flora, thereby leading to higher susceptibility to STI acquisition and transmission.^7^ ^22 ^Another noteworthy finding is that adolescents have vaginal immunityrelated mediators with a composition that differs from that of adults' mediators and consequently may respond differently to changes in the composition of the vaginal flora.^12^ In the present study, all cervicovaginal infections and alterations other than bacterial vaginosis were excluded, since they could act as potential confounders for cervicovaginal cytokine and sialidase levels.^23^ ^24 ^Our population was homogeneous between the two study groups, not only regarding sociodemographic variables but also in relation to the gestational age at the time of enrollment. 

It has been consistently shown in the literature that cervicovaginal IL-1 beta levels increase in response to bacterial vaginosis in non-pregnant^3^ ^15^ ^25 ^and pregnant women,^16^ which is in agreement with our current data on pregnant adolescents. IL-1 beta is crucial for the initial immune response to pathogens, but higher levels of this cytokine in the vaginal milieu increase the vulnerability to STI acquisition.^8^ Non-pregnant adolescents have significantly higher IL-1 beta levels, and this has been proposed as one of the mechanisms involved in the higher prevalence of STIs in this population.^12^

Data on vaginal IL-6 in the literature have shown that there is an association between increased levels of this cytokine in situations of aerobic vaginitis, but not of bacterial vaginosis.^13^ ^24 ^However, it has already been demonstrated that IL-6 levels are higher in pregnant women with bacterial vaginosis than in non-pregnant women with bacterial vaginosis.^16^ Recently, Balkus et al.^10^ failed to demonstrate any independent association between increased vaginal IL-6 levels and either pregnancy or bacterial vaginosis. However, they found that non-pregnant adolescents had higher IL-6 levels than those of adults.^12^ Our data provide the new information that IL-6 levels are even higher in pregnant adolescents with bacterial vaginosis.

Normally, IL-8 levels do not increase in situations of bacterial vaginosis,^15^ ^25^ which explains the unchanged number of leukocytes in vaginal smears, in comparison with normal flora.^26^ On the other hand, this scenario changes when pregnant women with bacterial vaginosis are evaluated in relation to non-pregnant women with the same floral alterations, such that significantly higher IL-8 levels are seen in pregnancy.^16^ According to Balkus et al., increased IL-8 levels were independently associated with both pregnancy and bacterial vaginosis.^10^ No change in cervicovaginal IL-8 levels has been associated with adolescence so far.^12^ Thus, based on data on the literature, we suggest that the increased cervicovaginal IL-8 levels reported here may have been due to pregnancy status rather than the younger age of the population. 

Bacterial sialidase is produced by several microorganisms associated with bacterial vaginosis^27^ and it degrades local immunoglobulins, thereby leading to impairment of the local immune response.^18^ In agreement with previous findings,^13-28^ the present study showed that sialidase was mostly detectable in situations of abnormal vaginal flora. This finding is particularly important in pregnancy, since detection of sialidase on vaginal samples has already been correlated with serious obstetric complications, such as preterm birth and preterm premature rupture of membranes.^19^ ^27^ Moreover, higher vaginal sialidase levels may increase the vulnerability to STIs during bacterial vaginosis, since they compromise the local defense against pathogens.^18^

The current findings highlight the importance of conducting further studies aimed at achieving better understanding of the vaginal immunity of adolescents. Considering that adequate treatment of bacterial vaginosis may be beneficial to this population by preventing STI acquisition and the risk of poor pregnancy outcomes, screening programs should be included during antenatal care. Further studies are still needed in order to develop and implement new strategies for STI prevention and for improvement of the reproductive health of this population. The high rates of abnormal vaginal flora and cervicovaginal infections reported here reinforce the need for screening for this particularly vulnerable population at prenatal services.

## CONCLUSION

Bacterial vaginosis is associated with a pronounced local inflammatory response and increased sialidase levels in pregnant adolescents, thereby contributing towards higher susceptibility of this population to STIs.
